# Effects of Everolimus in Modulating the Host Immune Responses against *Mycobacterium tuberculosis* Infection

**DOI:** 10.3390/cells12222653

**Published:** 2023-11-18

**Authors:** Anmol Raien, Sofia Davis, Michelle Zhang, David Zitser, Michelle Lin, Graysen Pitcher, Krishna Bhalodia, Selvakumar Subbian, Vishwanath Venketaraman

**Affiliations:** 1 College of Osteopathic Medicine of the Pacific, Western University of Health Sciences, Pomona, CA 91766, USA; anmol.raien@westernu.edu (A.R.); sofia.davis@westernu.edu (S.D.); michelle.zhang@westernu.edu (M.Z.); david.zitser@westernu.edu (D.Z.); michelle.lin@westernu.edu (M.L.); graysen.pitcher@westernu.edu (G.P.); krishna.bhalodia@westernu.edu (K.B.); 2 Public Health Research Center, New Jersey Medical School, Rutgers University, Newark, NJ 07103, USA; subbiase@njms.rutgers.edu

**Keywords:** *Mycobacterium tuberculosis*, everolimus, mTOR, autophagy, host-directed therapy

## Abstract

The phosphoinositide 3-kinase/protein kinase B/mammalian target of rapamycin (P13K/AKT/mTOR) pathway plays a key role in tuberculosis (TB) pathogenesis and infection. While the activity levels of this pathway during active infection are still debated, manipulating this pathway shows potential benefit for host-directed therapies. Some studies indicate that pathway inhibitors may have potential for TB treatment through upregulation of autophagy, while other studies do not encourage the use of these inhibitors due to possible host tissue destruction by *Mycobacterium tuberculosis* (*M. tb*) and increased infection risk. Investigating further clinical trials and their use of pathway inhibitors is necessary in order to ascertain their potential for TB treatment. This paper is particularly focused on the drug everolimus, an mTOR inhibitor. One of the first clinical trials sponsored by the Aurum Institute showed potential benefit in using everolimus as an adjunctive therapy for tuberculosis. Infection with tuberculosis is associated with a metabolic shift from oxidative phosphorylation towards glycolysis. The everolimus arm in the clinical trial showed further reduction than the control for both maximal and peak glycolytic activity. Compared with control, those receiving everolimus demonstrated increased lung function through forced expiratory volume in 1 s (FEV1) measurements, suggesting that everolimus may mitigate inflammation contributing to lung damage.

## 1. Introduction

Despite significant progress in disease control measures, vaccination, and antibiotic regimen treatment options, tuberculosis (TB) remains one of the leading causes of death in humans worldwide. TB, a bacterial infectious disease caused by *Mycobacterium tuberculosis* (*M. tb*), is acquired via inhalation of aerosols containing the pathogen, which most commonly affects the lungs [1]. However, TB can affect any part of the body, including the brain, gastrointestinal, reproductive, and musculoskeletal organ systems [1]. The outcome of initial *M. tb* infection manifests mostly in two forms: active disease and latent infection. In individuals with latent TB Infection (LTBI), the bacteria do not cause disease symptoms and the host is unable to spread the bacteria to others [2]. About 10% of individuals infected with *M. tb* develop active TB disease (primary TB) soon after infection, depending on both the host- and pathogen-derived factors [1]. In addition, LTBI cases may develop reactivation TB, in which the bacteria that were in a dormant state begin to actively replicate, causing symptomatic disease. About 90% of all TB cases are believed to be reactivation of LTBI, as compared to primary TB [1]. Thus, LTBI presents a serious challenge in the global fight towards TB eradication, as LTBI cases can unknowingly harbor the bacteria quiescently for many years.

Treatment for TB requires multiple antibiotics to be taken over the course of several months. The World Health Organization’s (WHO) policy of a directly observed therapy, short course (DOTS) strategy for TB has been shown to improve outcomes, especially in resource-poor countries [3]. This strategy involves standardized treatment protocols, supervision, and patient support, which is a broader form of the original DOTS that was first implemented. Between 2000 and 2015, absolute global mortality due to TB decreased by 22%, indicating that these newer strategies have been effective [3]. However, the incidence rates of TB have not declined as expected by experts in the field, pointing to the fact that one third of all TB patients are “unknown to the health system”, meaning that they do not seek care and do not receive treatment [3]. In addition, the global rise in Multi-Drug Resistant (MDR) and Extensively Drug Resistant (XDR) TB cases is posing significant hurdles in global TB management programs [3]. Thus, more progress is needed to accomplish complete eradication of all forms of TB.

Host-directed therapy (HDT) is emerging as a novel concept to improve the antibiotics-mediated treatment outcome for TB [4]. In this approach, repurposing of drugs that have already been approved by the US-FDA to treat other ailments are administered along with anti-TB medications. Several classes of such drugs, including autophagy inducers, phosphodiesterase-4 inhibitors, and anti-inflammatory drugs, have been tested for their ability to serve as effective adjunctive therapy for TB treatment [4]. In a recent phase 2 clinical trial, Everolimus, an autophagy inducer (mTOR inhibitor), was shown to improve clinical outcomes of anti-TB therapy among patients with pulmonary cavitary TB [5]. However, the precise effects of everolimus in modulating the host immune response during *M. tb* infection is not fully understood. In this systematic review, we analyze studies on everolimus-mediated immune regulation and discuss the effect of everolimus on autophagy pathways. in addition, we summarize the findings on preclinical and clinical trials as well as case studies in which everolimus has been tested.

## 2. Materials and Methods

To identify articles reporting the effects of everolimus on host immune responses to *M. tb* pathogenesis, a literature search was conducted during May to July 2023 in PubMed, which is the National Institute of Health–National Library of Medicine database. The search keywords included the following terms: “everolimus”, “*Mycobacterium tuberculosis*”, “autophagy”, “mTOR”, “pathogenesis”, and “host-directed therapy”. All articles published in the last 25 years were screened for relevancy and reviewed, and their citations were examined. Particular attention was placed on the year of publication for each of the articles to create a chronological order of findings relevant to everolimus and *M. tb*.

## 3. Epidemiology of *M. tb*

TB is the second leading infectious cause of death globally, behind COVID-19 and above HIV/AIDS [6]. In 2021, 1.6 million people died from TB [6]. The WHO considers Multi-Drug Resistant TB a public health crisis and threat to health security, as only 1 in 3 people with drug resistant TB accessed treatment in 2021 [6]. Tuberculosis infection has significant costs for health systems and countries as a whole, as well as for individual families affected by the disease. Worldwide, 50% of TB-affected households faced medical costs that were higher than 20% of their household income [6].

While tuberculosis infection is distributed across the globe, certain regions experience higher rates of infection compared to others. The highest rates of infection are seen in India, sub-Saharan Africa, Micronesia, and Southeast Asian islands [1]. The lowest rates are in Western Europe, the United States, Japan, Canada, and Australia [1]. China, Eastern Europe, Central and South America, and northern Africa have intermediate level rates [1]. In the United States, tuberculosis infection is seen predominantly in immigrant populations. Risk factors for developing active disease include immunocompromised state, chronic lung diseases, tobacco use, malnutrition, diabetes, alcohol use, IV drug use, indoor pollution, and end-stage renal disease [1]. Most notably, having HIV makes a patient 20 to 30 times more likely to develop active TB [1]. TB is the leading cause of death among HIV patients [6]. Each disease worsens the effects of the other, leading to worse outcomes for patients [6].

## 4. Pathophysiology of *M. tb*

*M. tb* is one of the five mycobacterial species that cause TB [1]. The bacteria possess multiple virulent factors that allow it to evade the body’s immune system. The cell wall of *M. tb* is waxy and has a high-lipid content, which allows it to resist antibiotics and survive in hostile conditions. Its capsule has a high mycolic acid content, which makes it a challenge for the body’s macrophages to destroy the bacteria [1]. In addition, the cord factor in the cell wall directly harms macrophages to protect the bacteria [1]. *M. tb* can resist oxidative stress via the action of catalase-peroxidase [1]. Despite these and other virulent factors, 95% of *M. tb*-infected individuals do not develop active TB because their adaptive immune system limits the progression of infection and contains it as LTBI [1]. The body’s ability to respond to the pathogen is dependent on the patient’s immune status, genetics, and the characteristics of the infecting bacilli [1].

At the cellular level, the innate immune cells, including the alveolar macrophages (AM), are the primary cells encountered by *M. tb* upon inhalation into the lungs [5]. In a naïve AM, *M. tb* can successfully mount an infection through phagocytic entry and intracellular survival within the AM. The *M. tb*-infected AM secretes proinflammatory cytokines and chemokines, which facilitate the extravasation of other immune cells, including the neutrophils, dendritic cells, and NK cells. Although the non-specific effector functions of the innate immune cells contribute to the control of bacterial dissemination, in most cases effective control of *M. tb* occurs only after the onset of adaptive immunity, which is mediated by T and B lymphocytes. The organized cellular structure at the site of *M. tb* infection, composed of innate and adaptive immune cells, constitutes the granuloma, which is a pathological hallmark of TB [5]. Within these granulomas, the intricate host–pathogen interactions play a crucial role in the progression of infection into active disease or establishment of LTBI.

## 5. Autophagy

Autophagy, also known as type II programmed cell death, is a cellular catabolically-driven process in which a double membrane-bound crescent-shaped vesicle termed a phagophore matures into an autophagosome that envelops long-lived proteins and damaged organelles [7,8,9]. The autophagosome then fuses with lysosomes, where its contents are released into the lysosome lumen and broken down into macromolecules [7,8]. in this way, autophagy serves as an effective means for the degradation of damaged cellular components and provides the cell with essential building blocks in both pro-survival and pro-death contexts [10]. Generally speaking, autophagy and apoptosis are primarily associated with pro-survival and pro-death, respectively, and the two modulate each other’s intensity of effects; however, they do not control each other, nor do they have the same underlying cellular mechanisms and activation [10]. Furthermore, autophagy plays a role in the basal turnover of cellular components, and could even avert apoptosis, which enhances the rates of cell survival. However, autophagy can play a role in pro-death contexts as well, which can occur either in concert with or independently of apoptosis, which usually arises with prolonged autophagy from excessive cellular stress [9].

## 6. Everolimus

In past years, everolimus (marketed as Afinitor, Zortress, and Afinitor Disperz) has generated excitement as a therapeutic agent for several ailments, including cancerous tumors and the prevention of organ transplant rejection [11,12,13,14]. Other reports indicate the possible use of everolimus as an anti-seizure medication due to its ability to modulate neuroinflammation [15]. Due to the aberrant activation of mammalian target of rapamycin (mTOR) signaling pathways during seizures, mTOR inhibition via rapamycin has been studied and found to be an effective anti-seizure therapeutic [16]. In search of other treatment agents, studies have been developed to compare the effectiveness of rapamycin and everolimus in the suppression of chronic seizure events. Interestingly, one such study indicated everolimus as a stronger therapeutic agent for the reduction of microglial associated inflammation recognized in Kainic Acid (KA)-induced seizure pathology. It is thought that inhibition of mTOR by rapamycin is key in this anti-inflammatory process [15]. Although somewhat limited in comparison to TB pathology, it should be noted that the mTOR pathways involved in KA-induced seizures were significantly more impacted with everolimus treatment compared to rapamycin. However, the current review is focused on investigating the potential positive effects of everolimus treatment with relation to pulmonary TB [17,18].

**Figure 1:**
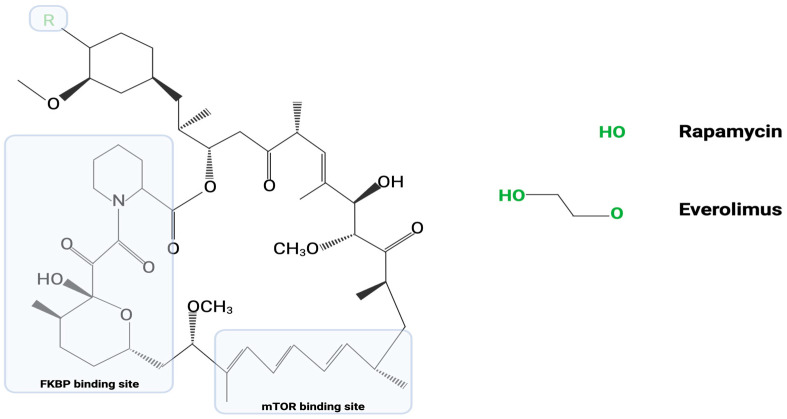
Represented in this image is the common chemical structure between rapamycin and everolimus. As discussed above, everolimus is an analog of rapamycin. The “R” group at carbon-40 represents the only variable region in their chemical structures.

As an analog of rapamycin, an established TB treatment, everolimus has similar structure, targets, and actions. Both agents contain an identical mTOR binding site and FKBP binding site. At the variable carbon-40 position, everolimus replaces the R-OH group of rapamycin with R-OCCOH [19] (Figure 1). The common mechanisms between the two agents primarily involve an ability to modulate intracellular mTOR messenger pathways [17]. Under standard conditions, the P13K/AKT/mTOR pathways lead to cellular inhibition of autophagy. This allows for cellular proliferation and growth; however, pathogens such as *M. tb* can exploit this autophagy inhibition and prevent normally programmed cell death of infected cells. In light of the ability of everolimus to inhibit these pathways and promote cellular autophagy, researchers have begun investigating the use of everolimus for the treatment of TB. Despite their similar mechanisms, large differences exist between rapamycin and everolimus. Everolimus was developed to provide an added bioavailability and a shorter half-life (15–35 h) compared to the half-life of rapamycin (58–63 h). Moreover, everolimus was developed in attempts to improve the low oral bioavailability seen in certain rapamycin treatment regimens [19,20].

Even though rapamycin and everolimus play very similar roles in the inhibition of mTOR pathways and stimulation of cellular autophagy, researchers have suggested that rapamycin may have a somewhat limited impact on TB. This may be due in part to the host cell metabolism via the cytochrome p450 3A4 (CYP3A4) enzyme, which might decrease the bioavailability of rapamycin [17,21]. These key pharmacodynamic and pharmacokinetic differences between rapamycin and everolimus suggest that the latter drug should be a seriously considered as a possible adjunctive therapy along with the current standard first-line anti-TB drugs, isoniazid, and rifampicin. Moreover, although TB treatment with rapamycin and everolimus would rely on fundamentally similar mechanisms, the key differences in mTOR pathway modulation between these two drugs implies a possible improvement in outcomes with everolimus use [15]. Furthermore, the continual development of multidrug resistant TB strains necessitates exploration of pharmaceutical alternatives for standardized treatment modalities [15,22,23].

## 7. P13K/AKT/mTOR Signaling in Autophagy

**Figure 2:**
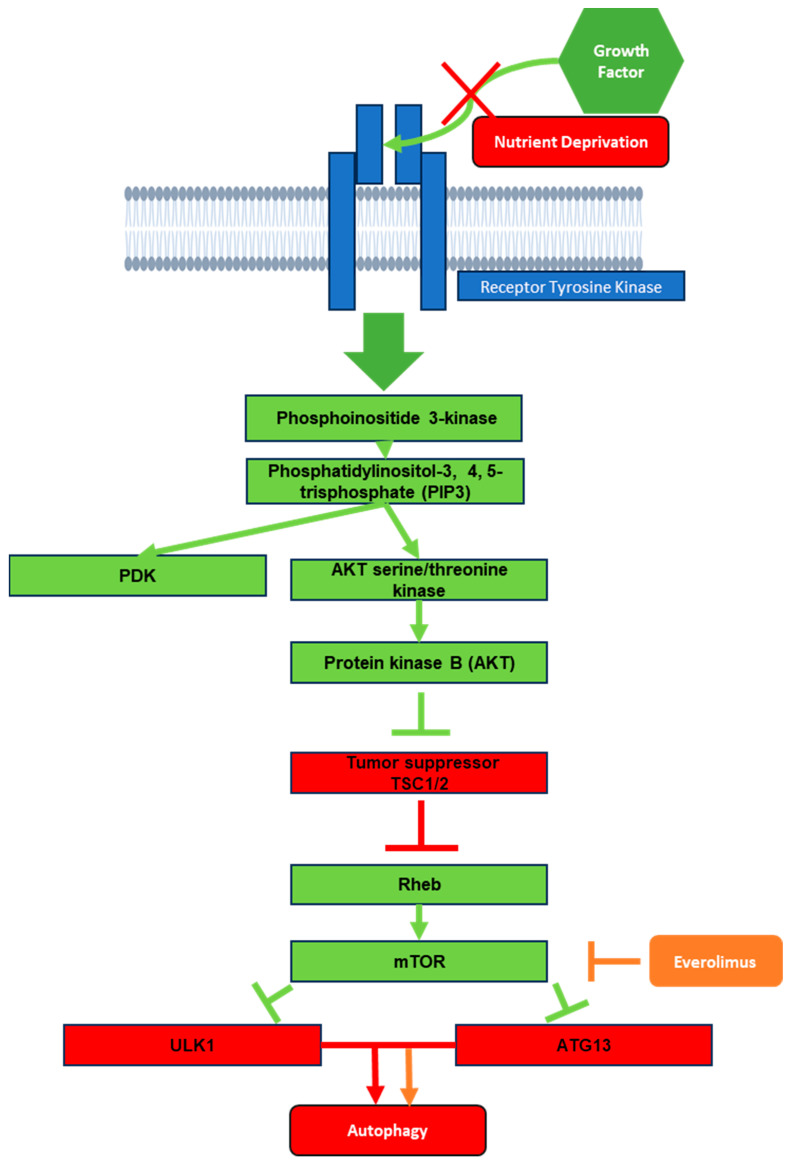
Schematic representation of mTOR pathway from the effects of growth factor down to autophagy. Green boxes represent activation signals during receptor tyrosine kinase exposure to growth factor. Red boxes represent what would remain activated in the absence of growth factor or in a nutrient deprivation state. Orange represents the effects of everolimus on the mTOR pathway.

The P13K/AKT/mTOR pathway, which is commonly associated with autophagy, offers multiple potential targets for HDT to treat TB. In this section, we focus on autophagy in the context of the P13K/AKT/mTOR pathway, where everolimus has a role as an allosteric inhibitor of mTOR (Figure 2). In the first step of phagophore nucleation, cellular stresses such as nutrient deprivation inactivate mTOR, a stress sensor, which results in the hypo-phosphorylation of downstream targets such as ULK1 (unc-51-like kinase 1) and autophagy related protein (ATG13) [7,24]. As a result, mTOR serves as a major inhibitory pathway of autophagy [25] through the P13K/AKT/mTOR axis, in which growth signals activate receptor tyrosine kinases (RTKs), which in turn activate phosphoinositide 3-kinase (PI3K) to generate phosphatidylinositol-3, 4, 5-trisphosphate (PIP3), which then recruits 3-phosphoinositide-dependent protein kinase 1 (PDK) and AKT serine/threonine kinase to activate protein kinase B (AKT) [26]. AKT in turn inhibits tumor suppressor tuberous sclerosis complex 1/2 (TSC1/2), a Ras homolog enriched in brain (Rheb) inhibitor. Loss of inhibition of Rheb leads to the activation of mTOR, causing inhibition of autophagy [26] (Figure 2). Lack of growth signals such as insulin led to the inhibition of mTOR by Rheb, which in turn activates autophagy, leading the cell to consume different organelles and proteins in order to recycle the building blocks and maintain cellular survival in a “starved” state. In addition, after nucleation, the phagophore is elongated and matured into an autophagosome via the PI3 kinases [9].

## 8. P13K/AKT/mTOR Signaling in TB

**Figure 3:**
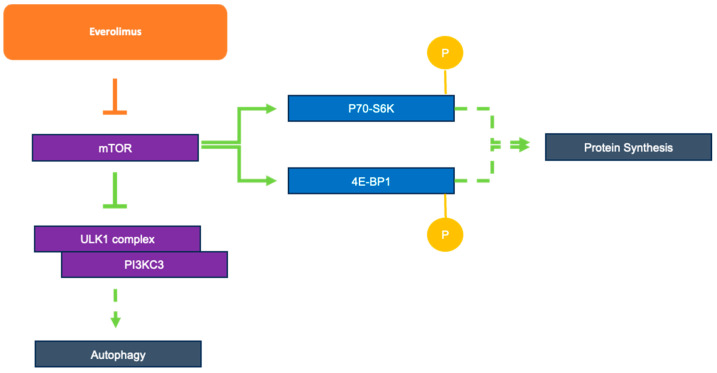
Role of everolimus as an mTOR inhibitor and mTOR’s downstream effects on autophagy and protein synthesis.

Active TB influences the P13K/AKT/mTOR signaling pathway; whether this pathway is stimulated or inhibited is complex, and may depend on the particular type of infected cells, such as peripheral blood mononuclear cell (PMBC) versus cell lines or T cells [27,28]. Patients with active TB exhibit activation of the P13K/AKT/mTOR pathway in CD14+ monocytes, as indicated by a study that found increased levels of the phosphorylated pathway targets p70 ribosomal S6 kinase (p70-S6K) and eukaryotic translation initiation factor 4E-binding protein 1 (4E-BP1) through Western blotting [27]. The same study found that the increased level of aerobic glycolysis in infected PBMCs and macrophages for rapid facilitation of the immune response was partly dependent on the AKT/mTOR pathway [27]. Interestingly, increased mTOR signaling prevented several components of autophagosome generation, particularly Unc-51-like kinase 1 (ULK1) complex and PI3KC3, from forming an initiation complex and reducing autophagy activity [29] (Figure 3). Considering that macrophages use autophagy as a key method for the destruction of *M. tb*, the pathogen remains able to negatively influence the regulatory pathway, allowing it to counteract this immune response and leading to active infection. These studies suggest that by targeting the P13K/AKT/mTOR pathway with, for example, an mTOR inhibitor, autophagy can be further activated in the host cells as a way to fight against the pathogen.

Inhibition of the P13K/AKT/mTOR pathway in T-lymphocytes and regulatory T cells (Treg) cells has been reported in patients with active TB [28]. During an active *M. tb* infection, there are significantly reduced levels of pathway components in cells, including CD3+AKT+ cells, CD3+p-AKT+ cells, CD3+mTOR+ cells, and CD3+p-mTOR+ cells in T lymphocytes and in CD4+CD25+FoxP3+Treg cells. This reduced activity leads to increased FoxP3+Treg cell activation, which aids in poor host control of infection and the potential for *M. tb* immune evasion [28]. Furthermore, inhibition of the P13Kδ/AKT/mTORC1 and mitogen-activating protein (MAP) kinase-interacting kinase (MNK) pathways was noted in primary human macrophages infected with *M. tb*. This inhibition resulted in increased secretion and gene expression of matrix metalloproteinase-1 (MMP-1), a peptidase critical for tissue remodeling during TB pathogenesis. In addition, P13K inhibition in infected macrophages upregulated the expression of proinflammatory markers, including cytokines, chemokines, and growth factors [30]. These studies, in contrast to those showing host-beneficial effects, indicate the potential for P13Kδ/AKT/mTORC1 and MAP kinase pathway inhibitors to increase the risk of *M. tb* infection.

Altogether, while the activity levels of the P13K/AKT/mTOR pathway during active TB remain debated, it is clear that these regulatory pathways play a crucial role in TB pathogenesis. While certain studies highlight the potential for host cell regulatory pathway inhibitors in TB treatment through upregulation of autophagy, others discourage these inhibitors in the context of host tissue destruction by *M. tb* and increased risk for disease progression. An analysis of clinical trials detailing the roles of specific P13K/AKT/mTOR inhibitors in TB infection is needed in order to evaluate their potential use as HDT for TB treatment.

## 9. Preclinical Trials of Everolimus

Research on the effects of everolimus in modulating the host immune response has demonstrated its potential as an adjunctive therapy for TB. As previously mentioned, everolimus is an inhibitor of the mTOR pathway and promotes autophagy in host cells [31]. In 2020, a study by Ashley et al. reported the efficacy of everolimus in a human granuloma model [22]. In this study, whole blood samples were taken from healthy human donors ranging in age from 18–65, and PBMCs were isolated for in vitro infection with the Erdman strain of *M. tb* for 8 days [22]. The study evaluated the host cell toxicity, intracellular survival of *M. tb*, and combinatorial effects of everolimus with the antibiotics isoniazid and pyrazinamide on the level of intracellular oxidative stress, autophagy, mTOR expression, and TNF-α production. This study revealed a significant difference in bacterial viability between 1 and 2 nM concentrations of everolimus-treated cells compared to the untreated control at 8 days post-treatment, indicating the potential of everolimus to induce suppression of *M. tb* growth in vitro [22].

The same study investigated the survival of *M. tb* after 8 days of exposure in in vitro granulomas with or without everolimus, isoniazid (INH), or pyrazinamide (PZA) and in granulomas treated with a combination of everolimus and INH or PZA. Compared to the control, a 60% reduction in bacterial growth was noted at 1 and 2 nm everolimus-treated human in vitro granulomas, supporting the effect of everolimus in reducing the survival of *M. tb* [22]. Interestingly, everolimus showed an additive antimycobacterial effect with INH and PZA, with overall reduction in the number of bacterial colony-forming units compared to the untreated control group [22]. Furthermore, intracellular oxidative stress was assessed by CellRox staining on the in vitro granulomas. The everolimus-treated granulomas, especially at the higher concentration (2 nM), showed reduced intensity of CellRox staining, indicating a downregulation in oxidative stress compared to the control [22]. In addition, autophagy levels were analyzed using a soluble protein marker, LC3B, found ubiquitously in mammalian cells, which is recruited from the cytosol during autophagy [32]. To determine the LC3B protein levels in the in vitro granulomas, an anti-LC3B antibody was tagged with phycoerythrin, a fluorescent protein that produces a red pigment. Treatment of *M. tb*-containing in vitro granulomas with 2 nM everolimus demonstrated a significant increase (about 50%) in the intensity of LC3B staining, which corresponds to increased autophagy in comparison to the sham-treated control group. Similarly, the INH plus everolimus treatment group showed a significant increase in levels of LC3B; interestingly, the INH-alone treatment group showed an increase in levels of LC3B compared to the untreated controls, showing that antibiotic treatment alone was effective in increasing autophagy marker expression in the *M. tb*-containing in vitro granulomas. However, although everolimus treatment significantly decreased the expression of mTOR compared to the control, as was expected, there was no clear trend in the expression of mTOR in granulomas treated with the antibiotics INH and PZA in conjunction with everolimus [32]. Therefore, the cellular mechanism of everolimus as a combinatorial therapy with front-line antibiotics appears more complex and requires further study.

TNF-α is a pro-inflammatory cytokine that plays an important role in the formation and maintenance of granulomas [33]. Everolimus treatment at 1 and 2 nM significantly reduced the levels of TNF-α compared to the control group, as did INH plus 2 nM everolimus compared to *M. tb*-containing in vitro granulomas only treated with INH. However, everolimus did not have any additive effect with PZA, which further suggests that there is complexity in the mechanism of everolimus action depending on the type of front-line antibiotics used along with everolimus in antimycobacterial studies.

Another preclinical study by Cao et al. evaluated the effects of everolimus on *M. tb*-containing in vitro granulomas generated from immune cells isolated from eight individuals with type II diabetes (T2DM) [34]. The study findings revealed that everolimus treatment significantly decreased the viability of *M. tb* in the in vitro granulomas at both 8 and 15 days post-infection, further supporting the potential of everolimus against *M. tb* infection in vitro [34]. A special group of cell types in the structure of a granuloma are the foamy macrophages, which overexpress lipid bodies and assist in establishment of LTBI and/or reactivating *M. tb* infection [35]. In this study, everolimus was found to significantly diminish the viability of *M. tb* after 72 h of infection in THP-1 macrophages. in addition, treatment with everolimus significantly decreased levels of lipid bodies inside THP-1 macrophages [35].

A double-blind clinical trial study was conducted in 2021 at Western University of Health Sciences to determine the effects of glutathione supplementation after 3 months of treatment in individuals with type 2 diabetes with the addition of in vitro everolimus on *Mycobacterium bovis* BCG strain [36]. The study included eleven subjects in the treatment arm supplemented with liposomal glutathione (L-GSH) and seven subjects in the control arm who received empty liposomes for 3 months [36]. the effects of the granulomatous responses against *M. bovis* BCG infection were investigated by sampling for the cytokines IFN-γ, TNF-α, IF-6, and IF-10 [36]. The results of the clinical trial showed that there was a statistically significant increase in the levels of IFN-γ, TNF-α, and IL-2 along with a notable increase of IL-6 in the everolimus treated L-GSH supplemented granuloma supernatant at 8 and 15 days post-in vitro BCG infection, compared to the control [36]. As everolimus is an mTOR inhibitor, it is supportive that such cytokines are upregulated during treatment with everolimus to modulate the host immune response to *M. tb*. In conclusion, these trials highlight the immune-enhancing effects of everolimus, with potential to modulate *M. tb* burden. The high global prevalence of T2DM poses a serious threat to the health of those with *M. tb* infection. Therefore, this study indicates the possibility of everolimus as an adjunctive therapy to help in modulating the immune response in those affected by TB and T2DM.

## 10. Case Study

In a case study, a 62-year-old East-Asian male oncological patient treated with everolimus for a neuroendocrine pancreatic neoplasia developed active TB twice in a year and a half [37]. The *M. tb* strain from the patient was tested with everolimus to determine its viability in an axenic culture and in a PMBC infection model. Everolimus’ direct anti-mycobacterial activity was tested against *M. tb* GM2659 and *M. tb* H37Rv strains in an axenic culture at 3.13 nM and 8.35 nM [37]. In this study, treatment with everolimus did not significantly reduce *M. tb* viability at either dose on either strain. Next, everolimus’ indirect antimycobacterial activity was tested in a PMBC infection model. PMBCs were infected with the *M. tb* GM2659 and *M. tb* H37Rv strains and treated with 3.13 nM and 8.35 nM everolimus, then were assessed for bacterial burden at 8 days post-infection. To validate the bacterial load assay, varying concentrations of *M. tb* H37Rv were plated on 7H11 solid growth medium containing everolimus at different concentrations (1 ng/mL, 3 ng/mL, 8 ng/mL, and 20 ng/mL). In addition, PMBCs treated with everolimus at different concentrations were challenged with *M. tb* H37Rv 4 days post-infection. There was no significant difference in *M. tb* growth on solid medium and no significant difference in CFU counting between the with and without everolimus conditions. These findings suggest that everolimus does not have a direct or indirect effect on *M. tb* viability at the administered concentrations [37].

The same study examined the effect of everolimus on the levels of intracellular reactive oxygen species (ROS). ROS levels were measured in THP-1 macrophages that were previously infected with *M. tb* H37Rv, GM2659, *Mtb*^H3^, and *Mtb*^EAI^ after treatment with everolimus at maximum therapeutic concentration. The results showed no increase in ROS production despite everolimus treatment [37]. These findings contradict those of previous studies that showed a beneficial effect of everolimus in controlling *M. tb*.

## 11. Clinical Trials

A phase 2 randomized open-label controlled trial was conducted in South Africa to determine the safety and preliminary efficacy of four potential HDT candidates for TB treatment [18,38]. One of the HDTs tested in this trial was everolimus. The study population included pulmonary cavitary TB cases with sputum-positivity for *M. tb*. For 180 days, all study participants received oral rifabutin-substituted standard TB treatment. However, participants who were randomized into the everolimus arm received an additional 0.5 mg/day of everolimus from day 1–112 of treatment [18,38]. The control group received standard anti-TB treatment [18,38].

The study looked at six measures, including safety, microbiologic effects in sputum and blood, PET/CT imaging, serum markers of inflammation, effects on *Mtb*-specific and general immune function, and pulmonary function [18,38]. In terms of safety, no treatment-emergent or treatment-attributable serious adverse events occurred in patients treated with everolimus, showing it to be a potentially safe and reasonably well-tolerated drug among patients with pulmonary TB [18]. Microbiologic effects of HDTs in sputum were evaluated via culture conversion. A successful sputum culture conversion consists of two consecutive negative sputum cultures in which no microbial growth occurs. Culture conversion is a prognostic marker used to indicate that a person is recovering from or cured of TB. In this phase 2 trial, no significant differences were seen in the culture conversion time between the everolimus-treated and placebo-treated groups [18]. The microbiologic effects in blood were measured by whole-blood mycobacterial activity, which can be used to investigate the immune cell response against TB [39]. Again, no significant difference was seen between everolimus and the control [40]. Glycolytic metabolism was assessed by (18)F-fluoro-2-deoxy-D-glucose PET imaging of participants. This was explored as a potential biomarker in the clinical trial. The precise mechanisms by which *M. tb* infection impacts glycolysis remains unclear. One study by Lachmandas et al. (2016) that was briefly discussed earlier suggested that there is a shift from oxidative phosphorylation towards glycolysis in macrophages infected with certain strains of *M. tb* [41]. However, Hacket et al. (2020) observed suppression of host glycolysis in macrophages infected with *M. tb* [42]. Everolimus specifically inhibits the mTORC1 complex, which can potentially downregulate glycolysis [43]. Nonetheless, the everolimus arm in the clinical trial mentioned above showed a reduction in both maximal and peak glycolytic activity on day 56 compared to the control group, although no difference in total glycolytic activity was observed [42]. The clinical trial additionally measured C-reactive protein (CRP) levels as a serum marker of inflammation. CRP is an acute-phase reactant produced by the liver and released during the innate immune response to infection. The level of CRP increases when there is inflammation in the body [44]. No significant difference in CRP measurements was noted between the control and everolimus-treated subjects [42].

Finally, this clinical trial assessed the lung function of subjects via spirometry. Despite favorable microbiological effects, long term sequelae from TB are common among cases treated with anti-TB drugs. Most of these cases develop bronchiectasis and lung fibrosis [37]. These are permanent conditions that impair lung function, directly affect quality of life, and contribute to increased long-term mortality [4]. Researchers have hypothesized that everolimus, which is a host immune modulator, may mitigate inflammation [37]. To assess lung function in a clinical trial, the forced expiratory volume in one second (FEV1) and forced viral capacity (FVC) data were collected via spirometry [45]. Compared with control, everolimus significantly increased FEV1 (as a percentage of predicted) at day 180 (6.56%, 0.18–12.95; *p* = 0.044). Although a 6% increase in FEV1 might seem modest, this is equivalent to an increase of 200 mL, and is considerably higher than the difference deemed to be clinically relevant in trials of chronic obstructive pulmonary disease [45].

Overall, this study showed potential benefits of using everolimus as adjunctive therapy in treating TB. Because there was no dose-ranging component to the trial and because pharmacokinetic analysis was not performed, the selected dose may have not been optimal for the indication or severity of disease [45]. Although the sample size was small and these results can only be considered preliminary, this study builds the framework to support future trials of HDT for TB while contributing to the knowledge of potential adjunctive therapy candidates [45]. The preclinical and clinical trials of everolimus are summarized in Table 1.

## 12. Conclusions

*M. tb* poses a great threat to human populations worldwide. Currently, TB treatment revolves around antibiotics, including INH in combination with rifampin, pyrazinamide, and ethambutol. HDT is a new modality of *M. tb* treatment that has gained traction in recent years as a way to augment the host immune response. Everolimus, a potential HDT, has shown promising effects in its ability to modulate the host immune response against *M. tb*. Several preclinical trials have demonstrated the ability of everolimus to impact the immune response by inhibiting mTOR and modulating autophagy during *M. tb* infection. To date, one successful clinical trial that has shown host-beneficial effects of everolimus as an adjuvant therapy with standard antibiotics for TB. However, in another case study, everolimus treatment did not have any effect on *M. tb* viability, and there was no increase in ROS production intracellularly in infected macrophages. These findings highlight the need for more studies in order to determine whether everolimus can decrease *M. tb* burden, especially in TB patients with comorbidities, as a way to further address the threat of *M. tb* globally. While the available data are insufficient to recommend everolimus as an adjunctive therapy, they support calls for further studies to explore everolimus and its ability to modulate the host immune response against *M. tb*.

## Figures and Tables

**Table 1:** Overview of studies regarding everolimus.

Author	Population	Everolimus Dose	Duration	Findings
Wallis, R.S. National Library of Medicine [18]	200	0.5 mg/day for 112 days	180 days	Everolimus was well tolerated, may also enhance the recovery of FEV_1_
Ashley et al., 2020 [22]	8 healthy subjects from ages 18–65	1 nM and 2 nM	8 days	Direct antimicrobial effect on in vitro *M. tb* granulomas. Decrease in TNF-α and downregulation in oxidative stress. Decreased expression of mTOR at 2 nM. No clear trend between everolimus treatment in conjunction with first line antibiotics.
Cao et al., 2021 [34]	8 T2DM positive subjects	1 nM	8 or 15 days	Everolimus restricts growth of *M. tb* within in vitro human granulomas Everolimus reduces intracellular survival of *M. tb* in THP-1 cells
To et al., 2021 [36]	11 T2DM positive subjects	1 nM	8 or 15 days	Statistically significant increase in IFN-γ, TNF-α, IL-2, and a notable increase of IL-6 post everolimus treatment in vitro.
Bianco et al., 2023 [37]	1 62-year-old East Asian male with history of pancreatic neuro-endocrine tumor	3 ng and 8 ng	8 days	No significant effect on viability or ROS production.

## Data Availability

No new data were created or analyzed in this study.

## Abbreviations

AKT, protein kinase B; ATG13, autophagy related 13, a protein coding gene; CFU, colony forming unit; CYP3A4, cytochrome p450 3A4; DOTS, directly observed therapy, short course strategy; FEV1, forced expiratory volume in 1 s; FKBP, FK506 binding proteins; FVC, forced vital capacity; HDT, host directed therapy; INH, isoniazid; KA, kainic acid; L-GSH, liposomal glutathione; MDR-TB, Multi-Drug Resistant tuberculosis; mTOR, mammalian target of rapamycin; PIP3, phosphatidylinositol-3, 4, 5-trisphosphate; PI3K, phosphoinositide 3-kinase; PZA, pyrazinamide; RTKs, Receptor Tyrosine Kinases; p70-S6K, p70 ribosomal S6 kinase; 4E-BP1, eukaryotic translation initiation factor 4E-binding protein 1; Treg, regulatory T cells; MAP kinase, mitogen-activating protein kinase; ULK1, unc-51-like kinase 1; XDR-TB, Extensively Drug Resistant tuberculosis.
